# Supplementary material for: What can entropy metrics tell us about the characteristics of ocular fixation trajectories?
Source: PLoS One. 2024 Jan 2;19(1):e0291823. doi: 10.1371/journal.pone.0291823 (PMC10760742; doi:10.1371/journal.pone.0291823)
Supplement: Attachment — Submitted filename: Review_Response.pdf [file pone.0291823.s001.pdf]

PONE-D-22-33922

What can Entropy Metrics tell us about types of fixations during video viewing?

PLOS ONE

Dear Dr. Melnyk,

Thank you for submitting your manuscript to PLOS ONE. I apologize for the prolonged review period. After careful consideration, we feel that it has merit but does not fully meet PLOS ONE's publication criteria as it currently stands. Therefore, we invite you to submit a revised version of the manuscript that addresses the points raised during the review process.

We thank the Academic editor for considering our manuscript and for providing a comprehensive and detailed review. We look forward to revising our manuscript. Our response to each point raised by the review is shown in red below.

Please submit your revised manuscript by May 25 2023 11:59PM. If you will need more time than this to complete your revisions, please reply to this message or contact the journal office at plosone@plos.org. When you're ready to submit your revision, log on to https://www.editorialmanager.com/pone/ and select the 'Submissions Needing Revision' folder to locate your manuscript file.

We have contacted the journal and requested a new deadline for our resubmission. Our new deadline is October 10th, 2023.

We look forward to receiving your revised manuscript.

Kind regards,

Patricia Wollstadt, Ph.D.

Academic Editor

PLOS ONE

Journal Requirements:

2. Thank you for stating the following in the Acknowledgments Section of your manuscript:

The study was funded by 3 grants to Dr. Komogortsev: (1) National Science Foundation, CNS-1250718 and CNS-1714623, www.NSF.gov; (2) National Institute of Standards and Technology, 60NANB15D325, www.NIST.gov; (3) National Institute of

Standards and Technology, 60NANB16D293.

However, funding information should not appear in the Acknowledgments section or other areas of your manuscript. We will only publish funding information present in the Funding Statement section of the online submission form.

We have deleted our Acknowledgments section.

Please remove any funding-related text from the manuscript and let us know how you would like to update your Funding Statement. Currently, your Funding Statement reads as follows:

(1) O.V.K., National Science 608Foundation, CNS-1250718 and CNS-1714623, www.NSF.gov;

(2) O.V.K., National Institute of 609Standards and Technology, 60NANB15D325, www.NIST.gov;

(3) O.V.K., National Institute of 610Standards and Technology, 60NANB16D293, www.NIST.gov;

the funders had no role in study design, data collection and analysis, decision to publish, or preparation of the manuscript.

This statement is fine.

Please include your amended statements within your cover letter; we will change the online submission form on your behalf.

3. We note that you have stated that you will provide repository information for your data at acceptance. Should your manuscript be accepted for publication, we will hold it until you provide the relevant accession numbers or DOIs necessary to access your data. If you wish to make changes to your Data Availability statement, please describe these changes in your cover letter and we will update your Data Availability statement to reflect the information you provide.

As of the date of this submission of our revised manuscript, an up to date version of our supplementary material, including eye-movement data, MATLAB code for computing entropy, and a spreadsheet containing all the final data used for the formal analysis in this manuscript is available at: https://digital.library.txstate.edu/handle/10877/16142.

Download “Supplementary_Materials.zip”

Reviewers' comments:

Reviewer's Responses to Questions

Comments to the Author

1. Is the manuscript technically sound, and do the data support the conclusions?

The manuscript must describe a technically sound piece of scientific research with data that supports the conclusions. Experiments must have been conducted rigorously, with appropriate controls, replication, and sample sizes. The conclusions must be drawn appropriately based on the data presented.

Reviewer #1: Yes

2. Has the statistical analysis been performed appropriately and rigorously?

Reviewer #1: Yes

3. Have the authors made all data underlying the findings in their manuscript fully available?

The PLOS Data policy requires authors to make all data underlying the findings described in their manuscript fully available without restriction, with rare exception (please refer to the Data Availability Statement in the manuscript PDF file). The data should be provided as part of the manuscript or its supporting information, or deposited to a public repository. For example, in addition to summary statistics, the data points behind means, medians and variance measures should be available. If there are restrictions on publicly sharing data—e.g. participant privacy or use of data from a third party—those must be specified.

Reviewer #1: Yes

4. Is the manuscript presented in an intelligible fashion and written in standard English?

PLOS ONE does not copyedit accepted manuscripts, so the language in submitted articles must be clear, correct, and unambiguous. Any typographical or grammatical errors should be corrected at revision, so please note any specific errors here.

Reviewer #1: No

We hope to be able to identify the issue the reviewer raises regarding our writing and correct it.

5. Review Comments to the Author

Reviewer #1: General comments

This manuscript provides a valuable overview of different entropy measures for fixation analysis during video viewing. Using the GazeCom database that includes gaze data for 18 video clips à 20 s each, the authors apply established entropy measures and evaluate their potential to characterize fixation trajectories. This approach and the summary of findings appears very useful for advancing gaze analyses in future studies focusing on viewing patterns in natural scenes. The overall outline of the manuscript is clear and technical information is mostly presented coherently. However, I have some suggestions how to improve the rationale structure and carve out the main insights more concisely. In addition, some general statements throughout the manuscript could be tuned down or phrased more cautiously.

Specific comments

Abstract: the abstract could be shortened significantly; in particular, the second half gives detailed information, e.g., on specific entropy features, that are hard to follow and need the introduction provided only later on. Thus, I suggest to focus just on the technical outline of the study and to

summarize the key conclusions on a more abstract level. The current concluding sentences seem quite fuzzy (Does the work really aims just at *this*, i.e., GazeCom, dataset? Is a focus on the differentiation between fixations and smooth pursuit really justified given the later discussion in the manuscript? Could the value for classification system be described more specifically?)

l.2ff the first sentence seems quite self-confident – I just encourage to rethink whether this strong claim should really be kept up

This statement is has been modified.

l.15ff maybe readers that do not have already advanced knowledge of chaos theory and entropy measures could profit from an overview table that characterizes cores measures; here and in the following sections a variety of technical terms is taken up that seems hard to follow without at least a rough classification.

We now include such a table in our revised introduction.

l.130ff this paragraph should be revised in order to give a concise introduction the following Methods section. Consider moving the information on the choice of entropy metrics and high-low analyses to the Methods section where the details are provided; I assume that here many readers might be just puzzled because it is not possible to understand the choice of methods. The general aim of the study (last sentence of the paragraph) could be phrased stronger. Also note that there are inconsistencies between statements in this paragraph and later on in the Methods section (see reasoning for choosing the GazeCom database).

We have edited this paragraph based on the reviewer's suggestion and our new data structure.

Methods:

I would appreciate a careful revision of the Methods section, including the Appendix material (additional note: the parallel offer of Appendix and Supplementals might be confusing). In the current version there is a plethora of figure in which the audience might get lost. Ideally it would be possible to re-evaluate which figures a really critical for getting the key findings. I would prefer less figures explained and documented in more detail; e.g., I think the figures in the complex facets of the Appendix' might not be self-explanatory.

In the original manuscript, figures and text describing the entropy metrics were included in the results section. All of this information is now in the Method section. The figures representing the High-Low analyses, which were in the Appendix of the original manuscript, are now in presented in the results section of the revision. In the original submission, we only analyzed the GazeCom, and with six entropies, and two pages per entropy (highest 36 fixations, lowest 36 fixations) there were 12 such figures. In the current version, we only show high-low pages that are informative, which, for GazeCom comes to 9 such figures. However, in the new version, we also analyze two additional datasets (Lund and OK Lab). These 2 datasets have 5 addition high-low figures. With these 14 figures plus the 10 figures in used in the method section to explain the entropy measures, we are submitting now a manuscript with 25 figures. We understand that this is a very unusual situation – most journals would never allow this many figures in one manuscript. However, to fully explain our methods and illustrate our high-low

analyses we simply need all of these figures. If the journal wanted us to move all of these 14 high-low entropy figures to an Appendix (either attached to the manuscript or available at a website) we would be glad to comply. We think our study would be easier to evaluate with all of the figures in the main manuscript. We leave the final decision about this to the review and the academic editor.

Table 2: Here you give the six selected measures, but the reasoning for the choice is only given in the Results section; the logical order should be improved.

In the original submission, we started with 15 entropy metrics (list all 15), and through a process of elimination, we reduced this number to six. Now we simply say:

In preliminary pilot studies, we started out with 15 entropy metrics (list all of them). Our preliminary results indicated that only six of these measures (list all 6) provided usable information about fixation trajectories. Therefore, below, we will discuss our results only for these six measures.

l.242ff the rationale for providing an elaborated description of some entropy measures does not get clear; *base entropy measures* seems to have only one subsection; the selection of different features is not motivated here (see first comment Methods)

As noted above, in the present manuscript we only describe and employ six entropy measures. We hope the reviewer finds this new presentation more clear.

l.334ff is it possible to explain the choice of *36* fixations?

For our high-low analysis, we wanted to show what the fixations with high entropy and low entropy look like. In our opinion, 36 fixations per figure was the most we could draw on one page clearly.

The statement that the manuscript cannot be understood without the Appendix' figures seems problematic – in my understanding an Appendix should provide additional information, not core information.

Based on this point raised by the reviewer, we now include all 14 of our high-low entropy figures in the main manuscript rather than in an Appendix.

I think it is necessary to prepare a comprehensible summary of relevant issues.

Personal references to co-authors and their appraisal appear unnecessary – the readers will form their opinion about the validity of the applied procedure.

The personal references to co-authors has been dropped in this revision.

Results:

The presentation of results seems not well-structured.

ll.356ff the reader would appreciate more than just *some idea*. The organization of the results section is not convincingly laid out and is also not reflected in the later paragraphs.

ll.377ff *that looked most promising* - sorry, that statement needs more substance

Table 6 it would be favorable if the order in which the measures are presented could be consistent in the Table and the text.

The results section of the revised manuscript is much clearer as it now only contains our high-low analysis.

Discussion:

The overall discussion of the provided insights could be more focused. Given the very stringent methods that the presented results are based on, it appears discrepant that conclusions remain rather arbitrary in the discussion section (e.g., *we don't really know*, *perhaps more detailed analysis of parameter choices….*, and so on). What are the main conclusions? What should the audience take along?

The discussion in the revised manuscript is completely different. It now emphasizes what entropy metrics tell us about fixation in three datasets. The meaning of high and low entropy are very dataset-specific.

The general criticism concerning GazeCom appears fundamental – I am not sure whether the authors really intend to punch the dataset in total, but at the moment it sounds a little bit like this. It would be interesting to know whether the authors tried to get into contact with GazeCom developers and have discussed these issues.

In the present resubmission, we can compare our results regarding the GazeCom dataset with the results from the more carefully classified Lund and OK Lab datasets. You can see that high and low entropy measures tend to find significant numbers of fixation misclassification in the GazeCom dataset but not the Lund and OK Lab datasets. A close review indicates that the Lund and OK Lab datasets were much more carefully classified. But this may not account for all of the difference. For the present analysis, the GazeCom dataset had 20,613 fixations, the Lund dataset had 145 fixations and the OK Lab dataset had 874 fixations. With more than 20 thousand fixations, the 36 highest and lowest fixations for GazeCom represent much more extreme cases than they do for the Lund and OK Lab datasets.
